# Regarding the role of HER2 overexpression on the survival in muscle-invasive bladder cancer

**DOI:** 10.1097/JS9.0000000000001091

**Published:** 2024-01-18

**Authors:** Wei Yang, Jinlin Liu

**Affiliations:** a Department of Clinical Laboratory, Zhejiang Provincial People’s Hospital Bijie Hospital, Bijie, Guizhou; b Department of Clinical Laboratory, South China Hospital, Medical School, Shenzhen University, Shenzhen, People’s Republic of China

*Dear Editor*,

We read Kim’s study^1^ with great interest. In this study, the author aims to investigate the effect of HER2 overexpression (HER2+) on the prognosis of muscle-invasive bladder cancer (MIBC), which includes 61 patients with clinically non-metastatic de novo MIBC and a subgroup of 33 patients who underwent immediate radical cystectomy (RC). Then, they use the Kaplan–Meier curve to evaluate the prognosis role of HER2 for 61 MIBC patients, and use univariate and multivariate Cox proportional hazards models to identify predictive factors for overall survival in all 61 MIBC patients. Despite definite results, we raise some concerns about the statistical results of this study.

First, the sample size of univariate and multivariate Cox proportional hazards is usually more than 15 times the confounder. The more confounders are analyzed, the more sample sizes are required. In Kim’s study, 6 confounders including age, sex, HER2 expression, radiologic findings, hydronephrosis, and pathologic findings of TURBT were included. However, the sample size was only 61 patients. Moreover, 7 confounders were found even in 33 RC subgroup patients. Thus, the statistical significance of univariate and multivariate Cox proportional hazards could result in inappropriate results in Khalid’s study.

Second, another important confounder may also be ignored when they use univariate and multivariate Cox proportional hazards model to identify predictive factors for 61 MIBC patients, that is, the commonly used chemotherapy drugs including cisplatin, methotrexate, vinblastine, doxorubicin or other radiotherapy. However, the drug use or radiotherapy history or their statistical significance in Khalid’s study were not listed, which could result in inappropriate results, although they excluded chemotherapy and radiotherapy patients when they analyzed the predictive factors for the 33 RC subgroup patients. However, they did not consider these confounders for all 61 MIBC patients.

However, although we felt the need to address these few points, we appreciate Kim’s paper^1^, which gives this opportunity to discuss the role of HER2 overexpression on survival in muscle-invasive bladder cancer.

## Ethical approval

Not applicable.

## Consent

Not applicable.

## Sources of funding

Not applicable.

## Author contribution

W.Y. and J.L.: discussed and wrote the manuscript.

## Conflicts of interest disclosure

We declare no conflicts of interest.

## Research registration unique identifying number (UIN)

Not applicable.

## Guarantor

Jinlin Liu.

## Data availability statement

This is a comment on a published paper; no data was presented in our comment, so the data statement is not applicable.

## Provenance and peer review

Not applicable.
